# RNAi of Sterol Δ24-Isomerase Implicated Its Involvement in Physalin Biosynthesis in *Physalis angulata* L.

**DOI:** 10.3389/fpls.2022.850711

**Published:** 2022-03-04

**Authors:** Jiao Yang, Jingyi Tian, Yuhui Yang, Yaru Zhu, Changfu Li, Yansheng Zhang

**Affiliations:** ^1^School of Environmental and Chemical Engineering, Shanghai University, Shanghai, China; ^2^School of Life Science, Shanghai University, Shanghai, China; ^3^Key Laboratory of Plant Germplasm Enhancement and Specialty Agriculture, Wuhan Botanical Garden, Chinese Academy of Sciences, Wuhan, China; ^4^College of Life Science, University of Chinese Academy of Science, Beijing, China

**Keywords:** *Physalis angulata*, physalin, 24-methyldesmosterol, 24ISO, campesterol

## Abstract

*Physalis angulata* is a renowned traditional Chinese medicine for the treatment of various conditions. Physalin is the major type of bioactive constituents conferring medicinal properties of *P. angulata*. Despite the medicinal importance, the pathways leading to physalin are largely unknown. In this study, we employed a transcriptomic approach to identify a *Pa24ISO* gene from *P. angulata*. Through heterologous expression in yeast, Pa24ISO was revealed to catalyze an isomerization reaction in converting 24-methylenecholesterol to 24-methyldesmosterol. Real-time PCR analysis showed that the abundance of *Pa24ISO* transcripts correlated with the accumulation pattern of physalin B in different tissues of *P. angulata*. A direct role of Pa24ISO in channeling of 24-methylenecholesterol for physalin B biosynthesis was illustrated by suppressing the gene in *P. angulata via* the VIGS approach. Down-regulation of *Pa24ISO* led to reduced levels of 24-methyldesmosterol and physalin B, accompanied with an increase of campesterol content in *P. angulata*. The results supported that 24ISO is involved in physalin biosynthesis in plants.

## Introduction

**Figure 1:**
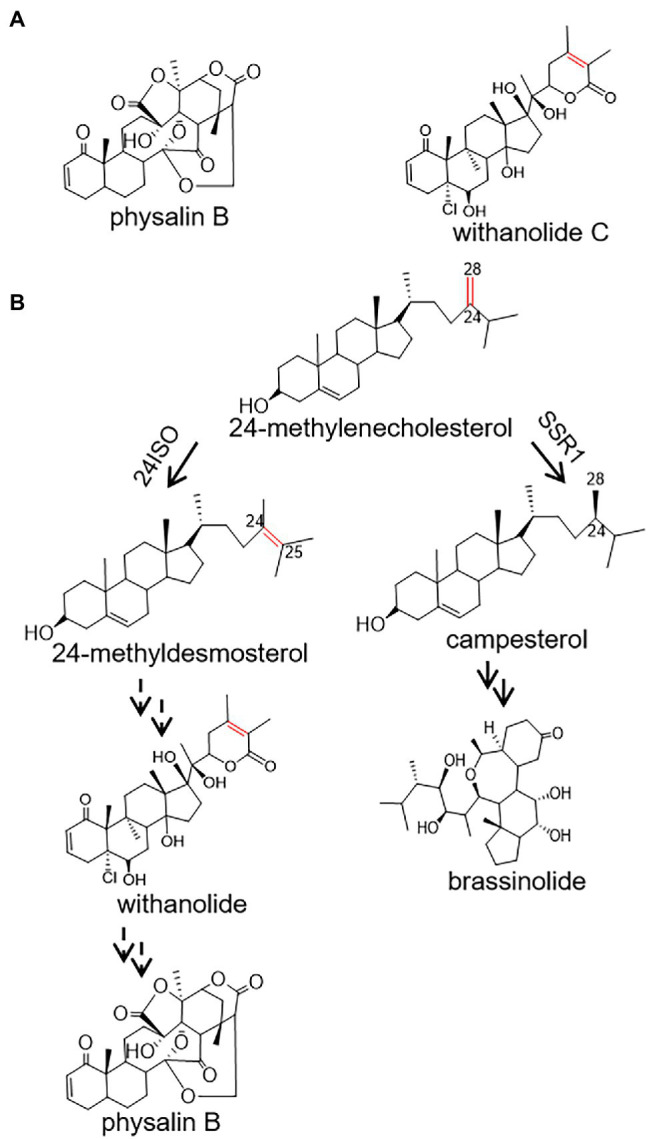
A proposed pathway for the biosynthesis of physalin B in *Physalis angulata* starting from 24-methylenecholesterol. **(A)** The structures of withanolide C and physalin B; **(B)** Proposed biosynthetic routes to physalin B and brassinolide. Biosyntheses of physalin B and brassinolide diverge at the level of 24-methylenecholesterol, which is channeled to the production of physalin B through withanolide *via* 24-methyldesmosterol under the activity of 24ISO, or of brassinolide *via* campesterol, catalyzed by SSR1.

*Physalis angulata* L, popularly known as Kuzhi in Chinese, has been prescribed for centuries for treatments of a variety of diseases, such as fever, malaria, liver disorders, and diabetes (Abe et al., 2006; Damu et al., 2007; Reyes-Reyes et al., 2013). Currently, crude extracts of *P. angulata* are reported to show antinociceptive (Choi and Hwang, 2003), anti-breast cancer (Hsieh et al., 2006), and anti-inflammatory activities (Bastos et al., 2008). The medicinal effects of *P. angulata* are mainly due to the presence of polyoxygenated steroids with an ergosterol backbone possessing a C-22, 26-lactone (Damu et al., 2007). Concerning modifications of their side chains, especially the cleavage of C13-C14 bond and the formation of a 16,24-carboncyclic bond, these steroid lactones can be classified as physalin and withanolide (Figure 1A; Huang et al., 2020; Xia et al., 2021). Of the *P. angulata* physalin, physalin B has received the most interest due to its diversely pharmacological activities. For example, it has been reported that physalin B showed significant activities in inhibition of several cancers, including breast (Wang et al., 2018) and prostate (Han et al., 2011) tumors.

Despite the pharmacological importance of physalin, the pathways leading to their biosyntheses are largely unknown. Physalin or withanolide are derived from a 24-alkyl sterol, and accordingly 24-methylenecholesterol is believed to be an intermediate in their biosynthetic steps (Pal et al., 2019). The 24-methylenecholesterol represents a branching point leading to biosynthesis of brassinolide *via* campesterol (i.e., 24-methyl cholesterol), catalyzed by sterol side chain reductase 1 (SSR1), or withanolide *via* 24-methyldesmosterol that is catalyzed by a sterol Δ^24^-isomerase (24ISO; Figure 1B; Knoch et al., 2018). The C24-C25 double bond in the intermediate 24-methyldesmosterol (Figure 1B) is retained in accumulated withanolide (see the structure of a representative withanolide, withanolide C, in Figure 1A). On the contrary, the C24-C25 double bond is not present in physalin-type compounds (see the structure of a representative physalin, physalin B, in Figure 1A). Probably because of this, Wang et al. have proposed an intermediacy role of campesterol (see its structure in Figure 1B) in physalin biosynthesis (Zhan et al., 2018). However, when taking analogous structures of physalin and withanolide (Figure 1A) into consideration, this study contemplated that physalin may be derived from withanolide, and this C24-C25 double bond may be rearranged later in the conversion stage from withanolide to physalin. If this hypothesis is followed, the enzyme 24ISO (Figure 1B) must involve in physalin biosynthesis.

To investigate whether 24ISO is involved in physalin biosynthesis *in vivo*, here, we reported the cDNA cloning and functional analysis of a *24ISO* gene, designated as *Pa24ISO*, from *P. angulata*. The biochemical activity of Pa24ISO was confirmed by heterologous expression in yeast. To further study its function *in vivo*, we are the first to develop a virus-induced gene silencing (VIGS) system for *P. angulata*. A direct role of Pa24ISO in physalin biosynthesis was discovered by silencing it in *P. angulata* through the VIGS approach. The *Pa24ISO* expression pattern also matched well with the accumulation of physalin B across different tissues.

## Materials and Methods

### Plant Materials

Seeds of *P. angulata* were harvested from the Langxi county, Anhui province of China in August 2018. The identity of the plant material was confirmed by Dr. Xiaodong Li at the Wuhan Botanical Garden, Chinese Academy of Science. *P. angulata* plants were grown in a growth chamber at 22°C under long-day (16 h of light/8 h of darkness) conditions. Transgenic plants were grown at an open field, located at the campus of Shanghai University, China.

### Identification of *Pa24ISO* and Phylogenetic Analysis

Using *Ws24ISO* (GenBank accession: AXG64144.1) as a query, a BLASTP analysis of our recently constructed *P. angulata* calyx-derived transcriptome (unpublished data) revealed the candidate *Pa24ISO*. Deduced amino acid sequences of Pa24ISO were aligned with the previously reported sequences of SSR1 and SSR2 (see their accession numbers in Supplementary Table 2) using ClustalW software embedded in MEGA7. A maximum likelihood tree was inferred in MEGA7 using bootstrap method with1000 replications. *Arabidopsis* DWF1 was used to root the tree.

### Establishment of a VIGS System for *Physalis angulata* and Generation of the *Pa24ISO*-Silenced Plants

The *PDS* (*phytoene desaturase*) gene, associated with the photobleaching phenotype, was used as a reporter gene to test the TRV-based VIGS system for *P. angulata*, The cDNA sequence of *PaPDS*, as shown in Supplementary Figure 1, was obtained from the *P. angulata* calyx transcriptome, and a 382 bp-fragment of *PaPDS* was amplified from the *P. angulata* calyx cDNA. For silencing the *Pa24ISO* gene expression, a 297 bp-region, which is specific to *Pa24ISO*, but is not conserved in the putative *PaSSR1* and *PaSSR2*, was selected (see their sequences in Supplementary Figures 2–4). All the primers used in this study are shown in Supplementary Table 1 in the Supporting Information. The TRV system constitutes the pTRV1 and pTRV2 vectors, which represent two RNA strands of the TRV virus system. The amplified DNA fragments of targeted genes were cloned into the pTRV2 vector *via* restriction cloning sites, yielding the pTRV2-PaPDS and pTRV2-Pa24ISO plasmids.

For down-regulating the expression of *PaPDS* gene, the pTRV2 (empty vector), pTRV1, and pTRV2-PaPDS were separately transformed into the *Agrobacterium* GV3101. The transgenic *Agrobacterium* strains were inoculated into LB medium supplemented with kanamycin at 50 mg/ml and rifampicin at 50 mg/ml at 28°C, and used for infection when OD_600_ value of the *Agrobacterium* culture reaching 1.5. For infiltration of the *P. angulata* leaf, the TRV1-contained *Agrobacterium* culture was mixed at a 1:1 ratio with the cultures containing pTRV2-PaPDS, as well as the empty vector pTRV2 as a control. To improve the infection efficiency, 0.01% Silwet L-77 was added into the *Agrobacterium* mixer. Four to six-leaf-stage plants were selected for the infection. The infected plants were kept in darkness for 48 h, and then grown at an open field at the campus of the Shanghai University, China. The phenotype was then evaluated after 6–8 weeks. The same procedure was applied to generate the *Pa24ISO*-silenced plants.

### Quantitative Real-Time PCR Analysis

The Real-time PCR (qRT-PCR) was performed on Bio-Rad CFX96™ Real-Time PCR instrument (Bio-Rad, Inc., United States) using TransStart Green qPCR SuperMix (Transgen). Primers used in this article are listed in Supplementary Table 1. The PCR program consisted of an initial step of 94°C for 30 s; 40 cycles of 94°C for 5 s and 60°C for 30 s; and then a dissociation stage of 95°C for 10 s, 65°C for 5 s and 95°C for 5 s. Each gene was assessed at least three biological replicates. The relative expression levels of the genes were calculated by the 2^-ΔΔCt^ method.

### Extraction of Physalin and UPLC–MS Analysis

Every 1 g of powdered *P. angulata* samples was extracted with 1.7 ml of 90% methanol by sonication for 30 min, and this procedure was repeated three times. To normalize variations between the different extractions, isofraxidin at a final concentration of 1.5 μg/ml was added as an internal standard. The solvent extracts were collected by centrifugation at 12,000 *g* for 20 min, and passed through a membrane filter (0.22 mm) prior to the Ultra Performance Liquid Chromatography–Tandem Mass Spectrometry (UPLC–MS) analysis.

One microliter of the extracts was injected for the UPLC-MS analysis. LC-MS analysis was performed using a Q-Exactive Focus mass spectrometer, coupled with a VanquishTM UPLC system (Thermo Fisher Scientific, Bremen, Germany) and a HESI source (Thermo Fisher Scientific). The column (100 mm × 2.1 mm, 1.8 μm) was used to separate the sample, the column temperature was 40°C, and the flow rate was 0.35 ml/min. The mobile phases contain 0.1% formic acid (solvent A) and acetonitrile (solvent B), and the solvent gradient is set as follows: 15% solution B (0–1.0 min), 20–25% solution B (2.0–13.0 min), 25–40% solution B (13.0–25.0 min), 40–90% solution B (25–26 min), 90% solution B (26.0–29.0 min), and 15% solution B (29.0–33.0 min). The MS detection was performed with a full MS mode. The parameters of the mass spectrometers were as follows: spray voltage, 3.2 kV; source capillary temperature, 320°C; sheath gas flow rate (nitrogen), 25 ml/min; Aux gas flow rate (nitrogen), 8 ml/min; Aux gas heater temperature, 30°C, Scan range 150.0–700.0 m/z.

### Heterologous Expression of *Pa24ISO* in Yeast

To optimize the expression of *Pa24ISO* in yeast, the full-length sequences of *Pa24ISO* were manually synthesized following the *S. cerevisiae* codon usage preference (see the optimized *Pa24ISO* cDNA sequence in Supplementary Figure 5), and inserted into a yeast expression vector pESC-HIS3 at *BamHI*/*SalI* sites, yielding the construct pESC-HIS3-Pa24ISO. The plasmid pESC-HIS3-Pa24ISO was transferred into a 24-methylenecholesterol-producing yeast strain YS11, which was recently developed by our group (Yang et al., 2021). As a negative control, the empty vector pESC-HIS3 was also transformed. The transgenic yeast colonies were selected on selection medium omitting histidine. Expression of the transformed gene was induced by 2% galactose.

To extract sterols from the transformed yeast cultures, yeast cells were pelleted from 2 ml of the yeast culture, broken with acid-washed glass beads, and extracted with 500 μl of 20% potassium hydrate in methanol (w/v) at 90°C for 2 h. When the saponified sample was cooled down, it was extracted with 2 ml of hexane for 30 min, and then was repeatedly extracted two times with another 1 ml hexane. The pooled hexane extracts were then dried in a vacuum freeze drier, and derivatized in 50 μl of N,O-bis(trimethylsilyl)-trifluoroacetamide (BSTFA) at 60°C for at least 1 h for sterol analysis by GC–MS.

### Phytosterol Extraction and Analysis by GC–MS

Phytosterol was extracted as described previously (Knoch et al., 2018) with small modifications. One gram of powered sample was extracted with 3 ml chloroform/methanol (2:1, v/v) at 75°C for 1.5 h. After removing the solvent, the dried residue was saponified in 1 ml 10% (w/v) KOH in MeOH for 1 h at 80°C. When the samples were cooled to room temperature, 500 μl of H_2_O and 500 μl of hexane were added, and extracted with shaking for 30 min. After centrifugation, the upper clear hexane extract was collected. The lower aqueous phase was then extracted twice with an additional 500 μl of hexane. The pooled hexane extracts were evaporated to dryness, and treated with 50 μl of the silylating reagent BSTFA [bis(trimethylsilyl)trifluoroacetamide]. The trimethylsilyl esters were dissolved in 80 μl of hexane, and a 1-μl sample was used for GC–MS analysis. For quantifying the sterol content, 40 μg of cholesterol (YuanYe, Shanghai, China) was added to each sample as an internal standard. GC–MS analysis was performed using a Shimadzu 2010 Plus GC machine coupled to a QP2020 Ultra mass spectrometer (Shimadzu Corporation, Canby, OR, United States). Samples were separated using a RTX-5 MS column (30 m × 0.25 mm × 0.25 mm) with helium being used as a carrier gas at a flow rate of 1.0 ml/min. The injection temperature was 280°C. The GC oven temperature program was as follows: 90°C for 1 min, then increased by 30°C/min to 300°C and hold at 300°C for 15 min. The mass of sterol compounds was detected in a select ion-scan mode with electronic ionization (70 eV), and monitored at multiple ions of *m/z* 343, 382, and 472 for campesterol, *m/z* 341, 365, and 470 for 24-methylenecholesterol, *m/z* 365, 386, and 470 for 24-methyldesmosterol, and *m/z* 329, 368, and 458 for the internal standard cholesterol.

## Results

### Identification of the *24ISO* Gene From *Physalis angulata*

**Figure 2:**
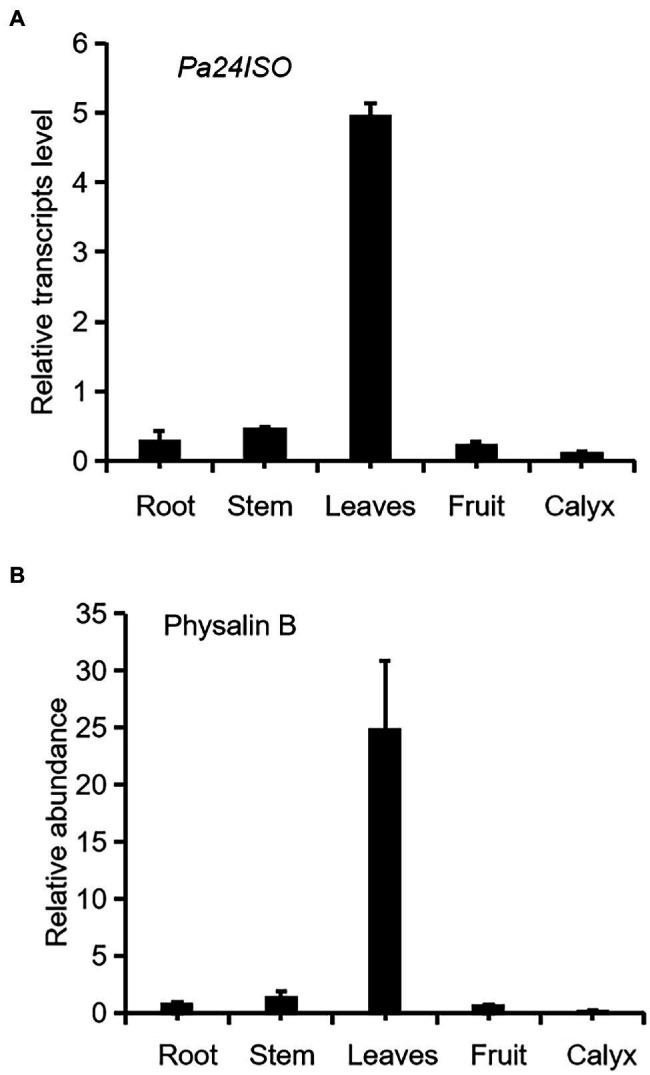
Transcript abundance of *Pa24ISO* **(A)** correlates with the accumulation pattern of physalin B **(B)** in different tissues of *P. angulata*. The tissues, including root, stem, leaf (the fifth leaf materials from the top), fruit, and calyx, were collected from four and half-month old *P. angulata* plants for the analyses. At this stage, the calyx was in the size of about 0.7 cm of diameter. Means and standard errors are shown (*n* = 3).

Firstly we have shown that physalin B accumulates to higher levels in leaf, compared to other tissues of *P. angulata* (Figure 2A). This result is different from a previous report (Huang et al., 2014), in which calyx was identified to be the physalin-accumulating site with the highest amounts. This discrepancy is probably due to the difference of the *P. angulata* cultivars utilized between this and that studies. The identity of physalin B was determined by comparing its MS/MS fragmented pattern (Supplementary Figure 7) with that published in a previous literature (Huang, 2014). A *Ws24ISO* gene was previously cloned from *Withania somnifera* (Knoch et al., 2018). In this work, using Ws24ISO as a query, we performed a BLASTP analysis against the transcriptome derived from *P. angulata* calyx that was recently constructed by our group. One unigene (ID number: PB.4414.9), hereafter refer to as Pa24ISO, was found to show the highest amino acid identity (96.0%) with Ws24ISO. A phylogenetic analysis shows that Pa24ISO closely clustered with Ws24ISO in the tree (Supplementary Figure 7), indicating that it is a potential 24ISO candidate. The expression of *Pa24ISO* was profiled in different organs with real-time RT-PCR. *Pa24ISO* transcripts were detected in the highest levels in the leaf of *P. angulata*, but in much less levels in other tissues (Figure 2B). The expression pattern of *Pa24ISO* matched well with the accumulation profile of physalin B across these tissues (Figure 2A).

### *Pa24ISO* Converts 24-Methylene Cholesterol to 24-Methyldesmosterol

To test the enzymatic function of Pa24ISO, the *Pa24ISO* gene was codon-optimized by manually chemical synthesis for preferential expression in *Saccharomyces cerevisiae*, and inserted into a yeast expression vector pESC-HIS3. As a positive control, the known gene *Ws24ISO* was also synthesized, and expressed in the same way as *Pa24ISO*. *Pa24ISO* or *Ws24ISO* was expressed in a 24-methylenecholesterol-producing yeast strain YS11, which was previously made by Yang et al. (2021). The yeast extracts were subsequently prepared, and analyzed by GC–MS for the presence of 24-methyldesmosterol. The negative control was prepared by transferring the empty vector pESC-HIS3 into the YS11 strain.

**Figure 3:**
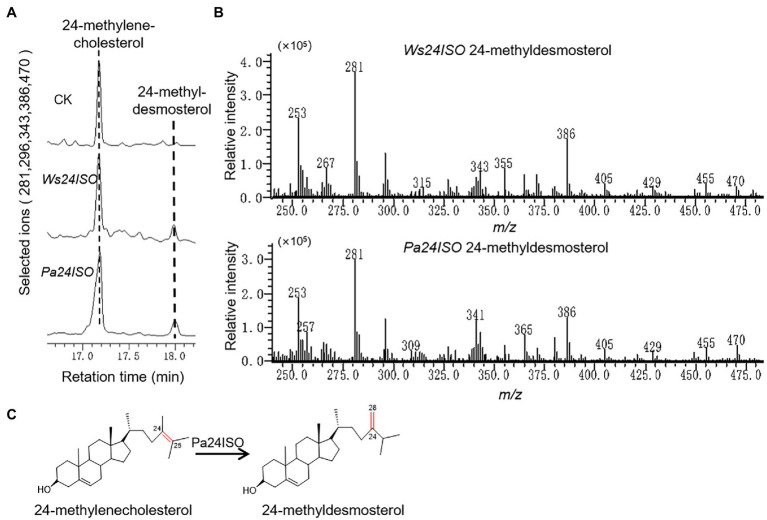
Enzymatic characterization of Pa24ISO in a 24-methylenecholesterol-producing yeast. **(A)** GC–MS chromatograms of yeast extracts from the yeast strain YS11 expressing *Pa24ISO*, *Ws24ISO* or empty vector. The 24-methyldesmosterol product was detected in the cells expressing *Pa24ISO* or *Ws24ISO* but not in the cells harboring the empty vector. **(B)** Mass spectra of the 24-methyldesmosterol product formed by Pa24ISO or Ws24ISO. **(C)** The reaction scheme showing the activity of Pa24ISO in converting 24-methylenecholesterol to 24-methyldesmosterol.

Cells expressing *Pa24ISO* showed a clear GC–MS product peak, which was missing from the empty vector control. This new GC–MS peak showed the same retention times (Figure 3A) and the same mass spectra with the 24-methyldemosterol product synthesized by Ws24ISO (Figure 3B). Thus, it is confirmed that the protein encoded by *Pa24ISO* is able to convert 24-methylenecholesterol to 24-methyldesmosterol (see the proposed reaction in Figure 3C).

### Development of a VIGS System for *Physalis angulata*

**Figure 4:**
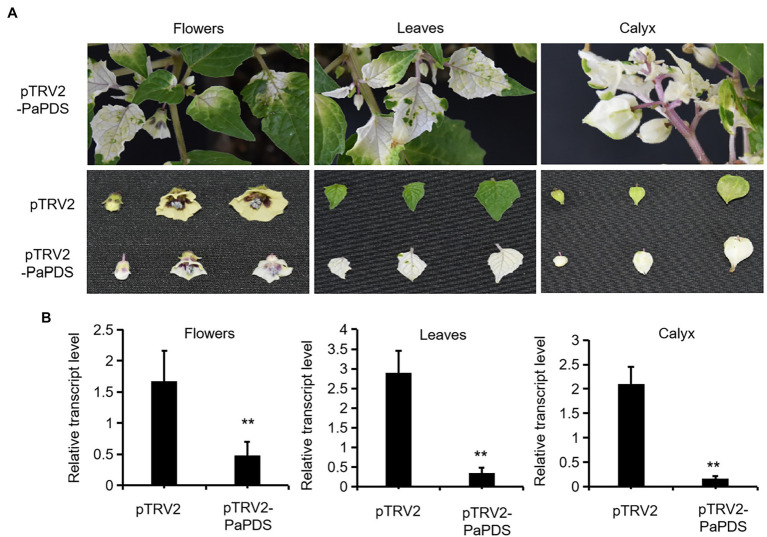
Silencing of *PaPDS* in *P. angulata* using the TRV2-based VIGS. **(A)** Representative photobleaching phenotype of flower, leaf and calyx tissues from the *PaPDS*-silenced plants. The upper row shows the intact *PaPDS*-silenced plants, and the below row shows the detached various tissues from the *PaPDS*-silenced plants, compared to those from the empty vector-transferred control. **(B)** qRT-PCR analysis of *PaPDS* in various tissues of *P. angulata* inoculated with pTRV2-PaPDS and the empty vector pTRV2. The error bars represent ± SE of three independent experiments (**p ≤ 0.01 by student t test).

To test functions of genes *in vivo* in *P. angulata*, it is necessary to establish a virus-induced gene silencing (VIGS) system for *P. angulata*, as there have been no reports of VIGS in this plant. For this purpose, the tobacco rattle virus (TRV; Burch-Smith et al., 2006) vector based VIGS system was employed, using *phytoene desaturase* (*PDS*; Cunningham and Gantt, 1998) as a reporter gene. The sequence of *P. angulata PDS* (*PaPDS*) was retrieved from the calyx transcriptome, which is available in our laboratory, and a 382 bp-fragment of *PaPDS* was selected as a target sequence. Six-to-eight week-old seedlings were infected with the *Agrobacterium* slurry containing the pTRV2-PaPDS vector or the empty pTRV2 vector as a control. At around 4 weeks post infiltration, a greenish–whitish variegated phenotype was observed in the upper leaves. With the growing of the infected seedlings, some of the newly emerging leaves exhibited a completely white phenotype (Figure 4A). Interestingly, at around 10–12 weeks post infiltration, a complete white phenomenon was also observed in the newly developed flowers and calyx of some of the infected plants (Figure 4A). We harvested the seeds that were generated from the plants showing completely white flowers or calyx, and grew them in soils to see whether the photobleaching phenomenon can be inherited in their offspring. However, they grew as wild type plants, and no photobleaching phenomenon was observed in their newly grown leaves.

To check whether the white phenotype correlated with the decreased *PaPDS* transcripts by VIGS, the *PaPDS* expression was monitored by real-time PCR. As shown in Figure 4B, the transcript levels of *PaPDS* were largely decreased in the photobleached leaf, flower and calyx, respectively, by the *PaPDS*-VIGS, compared with their corresponding empty vector controls. This result suggests that the TRV-based VIGS system can be used to suppress expression of genes of interest in *P. angulata*.

### The silencing of *Pa24ISO* Led to Reduced Levels of Physalin B in *Physalis angulata*

**Figure 5:**
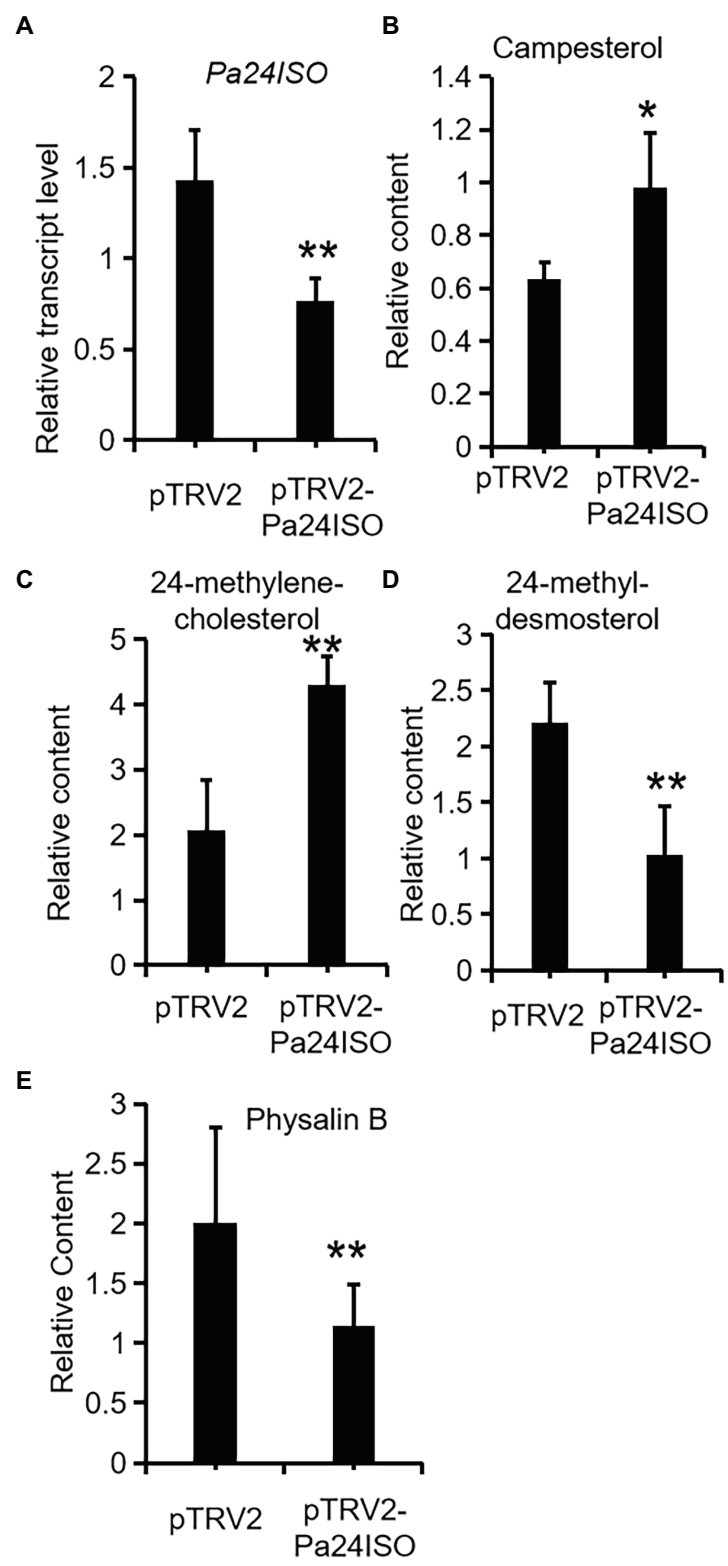
Downregulation of *Pa24ISO* resulted in reduced physalin B levels in *P. angulata*. The *Pa24ISO*-silenced plants (pTRV2-Pa24ISO) were compared to the empty vector-transferred plants (pTRV2), in analysis of expression of *Pa24ISO* **(A)**, and the relative abundances of campesterol **(B)**, 24-methylenecholesterol **(C)**, 24-methyldesmosterol **(D)**, and physalin B **(E)**. The error bars represent ± SE of three or six independent experiments (**p ≤ 0.01, *p ≤ 0.05; student *t* test).

After successfully establishing the VIGS system for *P. angulata*, we embarked experiments to silence the expression of *Pa24ISO in vivo*. Seven-week old plants were separately infiltrated with each of the *Agrobacterium* strains, which carried pTRV2-Pa24ISO, pTRV2-PaPDS, or pTRV2 empty vector, respectively. The pTRV2-PaPDS construct was utilized, as an indicator group, to monitor the silencing efficiency under different time lengths post the inoculation. Based on the visible phenomenon by pTRV2-PaPDS, the highest silencing efficiency was achieved at 8 weeks post the infection. Thus, after 8 weeks, the newly developed leaves from the infected plants transformed with the pTRV2-Pa24ISO, as well as the empty vector pTRV2, were harvested. To evaluate the silencing efficiency of *Pa24ISO*, quantification of the transcript level of *Pa24ISO* was performed with real-time PCR. In comparison with the control plants, which were infiltrated with the empty vector, the transcript level of *Pa24ISO* was down-regulated by 46% in the *Pa24ISO*-silenced plants (Figure 5A). It should be noted that the silencing of *Pa24ISO* also led to a decrease in the expression of a putative *PaSSR1*, however, it was not statistically significant (Supplementary Figure 8). Consistent with the reduced levels of *Pa24ISO* transcript, the *Pa24ISO*-silenced plants showed a sharp decline (54.3%) in the content of 24-methyldesmosterol, which was accompanied with increased accumulation of 24-methylenecholesterol and campesterol (Figure 5B and Supplementary Figure 8), when compared to the empty vector control samples. This result demonstrates that Pa24ISO is indeed the branch enzyme diverting the common substrate 24-methylenecholesterol toward biosynthesis of 24-methyldesmosterol-derived sterols, in competition with the campesterol-mediated carbon flux, as depicted in Figure 1B.

To investigate the effect of *Pa24ISO* silencing on biosynthesis of physalin, we quantified the content of physalin B in the newly developed leaves of the infected plants, through LC–MS/MS anlaysis. Physalin B appeared to be the major physalin compound in the *P. angulata* cultivar used in this study. The *Pa24ISO*-silenced plants displayed a 47% reduction in physalin B (Figure 5B), as compared with control leaves, indicating that *Pa24ISO* play a pivotal role in biosynthesis of physalin in *P. angulata*.

## Discussion

Physalin are the characteristic constituents identified in *P. angulata*, and they exhibit a broad spectrum of medicinal properties (Damu et al., 2007). The pathways leading to physalin are largely unknown. Since physalin is a class of 24-alkyl sterols, thus it is believed that they are undoubtedly synthesized from 24-methylenecholesterol. As depicted in Figure 1B, 24-methylenecholesterol is the branching point, which can be either directed into 24-methyldemosterol-mediated flux for withanolide *via* 24ISO (Knoch et al., 2018), or into campesterol-mediated flux for regular physterols *via* SSR1. Apparently, 24ISO competes with SSR1 at the level of 24-methylenecholesterol in the steps leading to withanolide production (Knoch et al., 2018). To date, there are no reports of physalin biosynthetic pathways in literatures. However, two possible pathways below were previously predicted by other groups: (1) physalins are biosynthesized from withanolides and (2) physalins are formed through the campesterol-pathway. The route 1 was recently proposed primarily based on a chemical viewpoint (Araki et al., 2021), in which withanolides can be transformed into compounds with a physalin-skeleton, through multiple oxidations. The route 2 was suggested, because that the withanolide content in *Withania somnifera* L was increased by feeding campesterol (Singh et al., 2014). For either route, there is no direct experimental evidence provided so far. For this reason, this project was designed with an aim to provide insight of which route that really occurs in nature for physalin production, through the silencing of *Pa24ISO* gene in *P. angulata*. This study is the first to isolate the *Pa24ISO* gene from *P. angulata*. Upon expression of *Pa24ISO* in a 24-methylenecholesterol-producing yeast strain YS11 (Yang et al., 2021), 24-methylenecholesterol was isomerized to 24-methyldesmosterol (Figure 3), implying that it is a functional 24ISO in *P. angulata*. The silencing of *Pa24ISO* was significant, with its transcript level being almost halved, as compared to the empty vector-transferred plants (Figure 5A). Physalin B was selected for the analysis, as it is present in high amounts in the *P. angulata* cultivar of this study, and could be reliably identified by comparing MS/MS fragmentation pattern with literature data (Huang, 2014). Compared to the empty vector controls, the *Pa24ISO* silencing led to significantly reduced level of physalin B (Figure 5). We also observed a 63% reduction for one unknown compound in the *Pa24ISO*-silenced plants (data not shown). Based on its mass spectra (Supplementary Figure 6), the molecular mass of this unknown compound matched that of withanolide C. If it was the withanolide C, the data that we have acquired is consistent with the previous finding, which clearly concluded that 24ISO is the first committed enzyme in the steps beyond 24-methylenecholesterol toward withanolide biosynthesis (Knoch et al., 2018). Therefore, it is highly likely that physalin is biogenetically synthesized from withanolide, and 24ISO is involved in physalin biosynthesis. An involvement of Pa24ISO in physalin biosynthesis could also be supported by a positive correlation of its transcripts with the accumulation profile of physalin B across different tissues of *P. angulata* (Figure 2). Taken together, these data corroborate that 24ISO plays a pivotal role in regulating physalin biosynthesis *in vivo*.

Upon the *Pa24ISO* silencing, physalin B and the intermediate 24-methyldesmosterol displayed a concurrently decreased accumulation pattern, whereas the change trend of campesterol was the reverse (Figure 5). This data further favors the proposal that physalin is biosynthesized through the 24ISO-catalyzed 24-methyldesmosterol pathway, while not through the SSR1-catalyzed campesterol (Figure 1B). The data of this study seem to be contradictory with a previous report (Singh et al., 2014), in which increased withanolide contents were found in *W. somnifera* plants fed with campesterol. However, campesterol is generally a precursor of C28-brassinosteroids (BRs; Bajguz et al., 2020), and feeding of campesterol would modulate levels of BRs, which, as signaling compounds (Kim and Russinova, 2020), may in turn regulate expression of withanolide biosynthesis genes. One might argue that the intermediate 24-methyldesmosterol is also the intermediate in the SSR1-catalyzed two-step conversions of 24-methylene cholesterol to campesterol (Figure 1B; Klahre et al., 1998; Takahashi et al., 2006), and physalin biosynthesis enzymes may also have access to the 24-methyldesmosterol produced by SSR1. However, although both 24ISO and SSR1 may co-localize to the same subcellular site (i.e., endoplasmic reticulum; Silvestro et al., 2013; Knoch et al., 2018), the 24-methyldesmosterol formed by SSR1 seems to be quickly subjected to the next reduction step instead of being released by SSR1. For example, expression of *Physalis alkekengi* SSR1 in a 24-methylenecholesterol-producing yeast strain led to the production of only campesterol with no trace of 24-methyldesmosterol (Knoch et al., 2018).

In short, this study provides experimental evidence to support that 24ISO is involved in physalin biosynthesis. Elucidation of the physalin biosynthetic pathway needs further isolation of the genes in the later steps beyond *24ISO*, such as the genes encoding cytochrome P450s, which probably catalyze multiple oxidations of the 24ISO product 24-methyldesmosterol to form the physalin.

## Conclusion

This study reports the isolation and functional analysis of a 24ISO-encoding gene (*Pa24ISO*) from *P. angulata*. Heterologous expression of *Pa24ISO* in yeast suggested that Pa24ISO is able to convert 24-methylenecholesterol to 24-methyldesmosterol. Silencing of *Pa24ISO* in *P. angulata* by the VIGS approach revealed a direct role of Pa24ISO in biosynthesis of physalin, such as physalin B at this case.

## Data Availability Statement

The original contributions presented in the study are included in the article/Supplementary Material, further inquiries can be directed to the corresponding author.

## Author Contributions

YZ received the funding to support this project. YZ and JY contributed to the writing. JY performed the experiments. CL provided the calyx transcriptome database and conducted the LC–MS/MS analysis. JT, YY, and YZ provided assistances in gene cloning, construct preparation, and chemical analysis. All authors contributed to the article and approved the submitted version.

## Funding

The work was jointly supported by a grant from the National Key R&D Program of China (2018YFC1706200) and the start-up funding grant to YZ from Shanghai University (N.13-G210-20-327).

## Conflict of Interest

The authors declare that the research was conducted in the absence of any commercial or financial relationships that could be construed as a potential conflict of interest.

## Publisher’s Note

All claims expressed in this article are solely those of the authors and do not necessarily represent those of their affiliated organizations, or those of the publisher, the editors and the reviewers. Any product that may be evaluated in this article, or claim that may be made by its manufacturer, is not guaranteed or endorsed by the publisher.
