# Supplementary material for: RNAi of Sterol Δ24-Isomerase Implicated Its Involvement in Physalin Biosynthesis in Physalis angulata L
Source: Front Plant Sci. 2022 Mar 4;13:850711. doi: 10.3389/fpls.2022.850711 (PMC8931419; doi:10.3389/fpls.2022.850711)
Supplement: Supplementary file 1 [file Data_Sheet_1.PDF]

Supplementary Material

RNAi of sterol Δ24-isomerase implicated its involvement in physalin biosynthesis in *Physalis angulata* L

Jiao Yang[1], Jingyi Tian[2], Yuhui Yang[2], Yaru Zhu[3,4], Changfu Li[2], and Yansheng Zhang[2*]

[1] School of Environmental and Chemical Engineering, Shanghai University, Shanghai, China, 200444;

[2] School of Life Science, Shanghai University, Shanghai, China, 200444;

[3] Key Laboratory of Plant Germplasm Enhancement and Specialty Agriculture, Wuhan Botanical Garden, Chinese Academy of Sciences, Wuhan, China,430074;

[4] University of Chinese Academy of Sciences, Beijing, China, 100049.

*** Correspondence:**
Yansheng Zhang
zhangys1@shu.edu.cn

Supplementary Tables.

Supplementary Table 1. Primers used in this study

The name of the primers	The sequences of the primers	Descriptions
Pa24ISO-F	GATGAGAAGGTGGCCACTGTT	For the qRT-PCR analysis
Pa24ISO-R	GCTTTCAGATTTCCGCGCAG	
PaPDS-F	GGAACTGAAAGTCAAGATGGTTGC	For the qRT-PCR analysis
PaPDS-R	GATGATGATAAGAATCAGCCTCC	
PaSSR1-F	CTTTCAAGCAGCGCCAGAAGGA	For the qRT-PCR analysis
PaSSR1-R	GCAATCATTCGCTCCTTGTCAATGTC	
PaGAPDH-F	GGTTGTCTCTA GCAGAGACCCTC	For the qRT-PCR analysis
PaGAPDH-R	CGC GACCTC GTG AGA GTA GTC	
Pa24ISO(EcoRI)-F	GGAATTCCATGTCAGATGAGAAGGTGGCCAC	For preparing the VIGS construct for the *Pa24ISO* gene
Pa24ISO(SacI)-R	CGAGCTCGATTCCTCATTCCAATAACCGTC	
PaPDS(EcoRI)-F	GGAATTCCCATTGCCATGTCAAAGGCACTTAAC	For preparing the VIGS construct for the *PaPDS* gene
PaPDS(SacI)-R	CGAGCTCGCCTTACATGTGACAGACATGT	

Supplementary Table 2. Accession numbers of the 24ISO, SSR1 and SSR2 homologs used in the construction of the phylogenetic tree. Sequences were extracted from the 1KP, SolGenomics, or GenBank database.

Gene name	1KP	GeneBank	SolGenomics
Nicotiana_benthamiana_SSR2-1			Niben101Scf00271g04029
Petunia_inflata_SSR2-1			Peinf101Scf00168g27017
Petunia_axilaris_SSR2			Peaxi162Scf00833g00024
Petunia_inflata_SSR2-2			Peinf101Scf00838g02001
Capsicum_annuum_SSR2		XP016559313	
Solanum_melongena_SSR2			Sme2.5_04335.1
Solanum_incanum_SSR2			SIUC37455_TC01
Withania_somnifera_SSR2		MH184047	
Physalis_alkekengi_SSR2		MH184043	
Solanum_tuberosum_SSR2		BAQ55275	
Solanum_lycopersicum_SSR2		NP001306251	
Solanum_pennellii_SSR2		XP015065843	
Solanum_sisymbriifolium_SSR1	NMDZ2018758		
Solanum_sisymbriifolium_SSR2	NMDZ2019294		
Datura_metel_SSR1	JNVS2005019		
Datura_metel_SSR2	JNVS2005020		
Datura_metel_24ISO	JNVS2005090		
Brugmansia_sanguinea _SSR1	AIOU2071007		
Brugmansia_sanguinea_24ISO	AIOU2011639		
Solanum_ptychanthum_SSR2	DLJZ2057967		
Solanum_dulcamara_SSR2	GHLP2059343		
Solanum_xanthocarpum_SSR2	LQJY2005212		
Solanum_lasiophyllum_SSR2	DLAI2007236		
Atropa_belladonna_SSR1	BOLZ2004405		
Lycium_barbarum_SSR1	LWCK2000969		
Nicotiana_sylvestris_SSR1	MKZR2007100		

Solanum_dulcamara_SSR1	GHLP2011409		
Solanum_xanthocarpum_SSR1	LQJY2017653		
Solanum_ptychanthum_SSR1	DLJZ2010546		
Solanum_lasiophyllum_SSR1	DLAI2017564		
Arabidopsis_thaliana_DWF1		NP188616	
Solanum_aethiopicum_SSR1			SAUC12718_TC01
Solanum_aethiopicum_SSR2			SAUC86019_TC01
Solanum_pennellii_SSR1		XP015065796	
Solanum_lycopersicum_SSR1		BAQ55272	
Solanum_tuberosum_SSR1		NP001275194	
Withania_somnifera_SSR1		MH184045	
Physalis_alkekengi_SSR1		MH184046	
Physalis_peruviana_SSR1		MH184044	
Petunia_axilaris_SSR1			Peaxi162Scf01123g00017
Petunia_inflata_SSR1-1			Peinf101Scf01614g01040
Petunia_inflata_SSR1-2			Peinf101Scf01590g03007
Capsicum_annuum_SSR1		XP016558623	
Nicotiana_tabacum_SSR1-2		XP016462759	
Nicotiana_tomentosiformis_SSR1		XP009624735	
Nicotiana_attenuata_SSR1		XP019231954	
Nicotiana_tabacum_SSR1-3		XP016467629	
Nicotiana_benthamiana_SSR1-2			Niben101Scf02156g03023
Nicotiana_benthamiana_SSR1-1			Niben101Scf04964g02005
Petunia_inflata_24ISO-2			Peinf101Scf00464g06012
Petunia_inflata_24ISO-1			Peinf101Scf00464g01010
Petunia_axilaris_24ISO-1			Peaxi162Scf00695g00218
Petunia_axilaris_24ISO-2			Peaxi162Scf00312g00221
Withania_somnifera_24ISO		MG184042	

Physalis_alkekengi_24ISO		MH184048	
Physalis_peruviana_24ISO		MH184049	
Capsicum_annuum_24ISO		XP016575706	
Nicotiana_tomentosiformis_SSR2		XP009624490	
Nicotiana_tabacum_SSR2-1		XP016452205	
Nicotiana_attenuata_SSR2		XP019235127	
Nicotiana_benthamiana_SSR2-2			Niben101Scf03969g04003
Nicotiana_tabacum_SSR2-2		XP016452205	
Nicotiana_sylvestris_SSR2	MKZR2007099		

Supplemental Figures

atgccccaaattggacttgtttctgctgttaacttgagaatccaaggtaattcagcttatctttggaggtcaaggtcttgtttgggaactgaaagtcaaga
tggttgcttgcaaaggaattctttatgttttggtggtagcgaatccatgggtcataagttaaaaagccctactccccttgccatgaacagaagattggct
aaggacttgcggcctttaaaggtagtttgcattgattatccaaggccagagctagacaatacagttaactatttggaggctgcattcttatcatcatcatt
ccgaacttctcctcgcccaactaaaccattggagattgttattgctggtgcaggtctgggtggtttgtctacagcaaaatatttggcggatgctggtca
caaaccgatactgctggaggcaagggatgttctaggtggaaaggttgccgcatggaaagatgatgatggagattggtacgagactggtttgcaca
tattctttggggcttacccaaatatacagaacctgtttggagaattagggattaacgatcgattgcagtggaaggagcattcaatgatatttgcaatgc
ccagcaagccgggagaattcagccgttttgatttccccgaagctttacctgctcctttaaatggaattttggccatcctgaaaaacaatgaaatgctta
catggccagagaaagtcaaatttgcaattggactgttgccagcaatgcttggagggcaatcttatgttgaagctcaagacggaataagtgttaagga
ctggatgagaaagcaaggcgtgccggatagggtgacagatgaggtgttcattgccatgtcaaaggcacttaactttataaaccccaatgagctttca
atgcagtgcatcttaatcgcgttgaacagatttcttcaggagaaacatggttcaaaaatggcctttttagatggtaatcctcctgagaggctttgcatgc
cgattgttgaacatatcgagtcaaaaggtggccaagtaagactaaactcacgaataaaaaagattgagctgaatgccgatggaagtgtcaaatgttt
tatcctgaacgatggtagtacaattaagggagatgcttttgtgtttgccactccagtggatattctcaagcttcttttgcctgaagactggaaagagattc
catatttccaaaagctggagaagttagtcggcgtacctgtgataaatgtacatatatggtttgacagaaaactgaagaacacatatgatcatttgctttt
cagcagaagttcactgctcagtgtgtatgctgacatgtctgtcacatgtaaggagtattacaaccccaatcagtctatgttggaattggtttttgcacct
gcagaagagtggatttctcgcagcgactcagaaattattgatgctacaatgaaggaactagcaacgctttttcctgatgaaatttcggcagatcagag
caaagcaaaaatcttaaagtatcatgttgtcaaaactccaaggtctgtttataaaactgtgccaggttgtgaaccctgtcggcccttgcaaagatcgcc
tatagaggggttttatttagctggtgactatacaaaacagaaatacttagcttcaatggaaggtgctgttttatcgggaaagctttgtgcacaagctatt
gtacaggattatgagttacttgttggccggagccagaaggtggaggtggaagcaagcttagtttaa

Supplemental Figure 1. The cDNA sequences of *PaPDS* retrieved from the calyx transcriptome of *P. angulata.* The part highlighted in green colors was selected as the targeted fragment for silencing the *PaPDS* expression by the VIGS.

ATGTCAGATGAGAAGGTGGCCACTGTTCGTCCCAAGAGGGAGACGCGGTTGGTGGACTT
TCTTGTCCAATTCAGATGGATCGTTGTTATATTCTTCGTCCTTCCTTTCTCGTGCTTGTACT
ACTTCTCCATATACCTAGGGGATGTTAGATCTGCGCGGAAATCTGAAAGCCAGCGTAAG
AAGGAACACGAAGAAAACATTAAAGAGGTTGTGAAGCGCCTTGCACAGAGAGATGCGT
CAAAGGATGGCCTTGTTTGCACAGCCAGGCCTCCCTGGACGGTTATTGGAATGAGGAAT
TGTGACTATAAGCGTGCTCGTAGTTTCAAAGTTGATCTTTCCAAGTTTAGAAATATTCTT
GAAATCAACAAGGAACGAATGGTTGCAAAAGTTGAGCCTCTTGTCAATATGGCCCAAAT
CTCTAGAGCTACTATACCCATGAATCTTTCGCTTGCAGTTATTGGCGAGCTTGATGATCT
GACTGTTGGTGGTTTGATCAATGGATTCGGGATTGAAGGAAGCTCTCACATCTATGGATT
GTTCTCTGACACAATAGTGGCACTCGAGGTTGTTCTGGCTGATGGACGGGTGGTTAGAG
CTACAAAGGACAACGAGTACTCCGATCTTTTCTATGCTATCCCATGGTCACAGGGGACA
TTGGGGCTTCTTGTTTCAGCCGAAATTAAGCTCATACCTGTGAAGGAATACATGAAGCTT
ACCTACAAACCTGTAACTGGTAATCTAAAAGAGCTAGCACAGGCCTTTGCAGATTCCTTT
GCACCGAGAGACGGAGACCAGGATAATCCTAGTAAAGTTCCAGAGTTTGTTGAAGGCAT
GATTTACAGTCCCACGGAAGGTGTTATGATGACGGGTAGATACGCTTCGACAAAAGAGG
CCAAGCAAAAGGGCAACGTAATCAATAGTATCGGTTGGTGGTTCAAGCCATGGTTTTAC
CAGCATGCTCAAACTGCACTGAAAAGAGGGGAGTTTGTAGAGTACATTCCAACTAGAGA
TTACTGTCACAGGCACACAAGATCCATCTACTGGGAAGGCAAATTAATTCTTCCGTTTGG
GGATCAGTTCTGGTTTAGGTATCTCTTTGGATGGATGATGCCTCCCAAGGTTGCTGTGCT
CAAGGCCACTCAAAGTGAATATATCAGGAATTATTACCATGATAATCATGTCATTCAGG
ATTTGCTCGTTCCTCTTCACAAGGTCGCTGATACTCTCGAGTGGGTCCACCGCGAGATGG
AGGTATATCCTGTGTGGCTCTGCCCACACAGAATCTACAAGCTGCCTGTGAAACCTATG
ATCCATCCTGAACCAGGATTCGAGGCACACAAGAGACAGGGCGACACTAAATATGCCC
AAATGTATACTGATGTTGGCGTCTACTATGCTCCTGGAGCTGTCCTAAGGGGTGAGCCCT
ATGATGGCGCAAAGAAATGCCATGACGTGGAGATTTTTCTAATCGAAAACCATGGATTC
CAGCCTCAATATGCGGTGACTGAGCTATCAGAGAAGAACTTCTGGAGGATGTTTGATGC
AACCTTATACCAGCAATGTAGAAAAAAGTACAAAGCCATTGGAACATTCATGAATGTGT
ACTATAAATGCAAGAAAGGGAAGAAGACGGAGAAGGAAGTGCAAGAAGCTGAGCAAG
AGAAAGCTGAGCTTGAAACTCCCGAGGTCGATGAGCCCGCGGATTGA

Supplemental Figure 2. The cDNA sequences of the *Pa24ISO* retrieved from the calyx transcriptome of *P. angulata.* The sequences highlighted in red colors are selected for silencing the *Pa24ISO* expression by the VIGS.

ATGTCAGATCTTGAGGCTCCCCTTCGCCCTAAGAGGAAGAAAAACATGGTTGACCTTCTT
GTCCAATTCAGATGGATCGTCGTTATCTTCGTTGTGCTTCCTCTCTCTTTCCTGTATTATTT
CTCCATATATCTTGGGGATGTTAGATCCGATTGCAAATCTTTCAAGCAGCGCCAGAAGG
AGCATGATGAAAATGTTAAAAAGGTTGTAAAGCGTCTTAAGGAGAGGAATGCATCTAA
GGATGGTCTTGTCTGCACAGCTAGAAAGCCCTGGGTTGCTGTTGGAATGAGAAATGTGG
ACTACAAGCGTGCTCGTCATTTCGAAGTTGATCTTTCTCCATTTAGAAATATTCTTGACA
TTGACAAGGAGCGAATGATTGCTAGAGTTGAGCCTCTAGTCAATATGGGCCAAATCTCT
AGAGTTACTGTTCCTATGAATGTTGCTCTGGCAGTTGTTGCTGAGCTTGATGATCTAACT
GTTGGTGGTCTGATCAACGGCTACGGGATTGAAGGAAGTTCTCACCTTTATGGCCTCTTC
TCAGACACTGTTGTGTCATATGAAGTTATCCTAGCAGACGGGCAGGTAGTTAGAGCTAC
AAAGGACAACGAATATTCCGATCTTTTCTATGCTATACCATGGTCTCAAGGGACTCTGGG
GCTTCTTGTTTCAGCAGAGATCAAGCTCATACCGATCAAGGAATACATGAAACTCACTT
ACAAGCCTGTAGTTGGTAATCTGAAAGAGATTGCACAGGCTTATATAGATTCTTTTTCAC
CTAGAGATGGGGATCAGGATAATCCTGCTAAGGTTCCATACTTTGTAGAAACCATGGTG
TACACTCCCACAGAAGCTGTTTGCATGACTGGTAGATACGCTTCAAAAGAAGAGGCCAA
GCAGAAGGGAAATGTGATCAACAATGTTGGTTGGTGGTTCAAAACCTGGTTTTACCAGC
ATGCTCAAACTGCACTAAAGCGGGGGGAATTCATAGAGTACATTCCAACTAGGGAATAC
TACCACAGGCACACAAGATGCTTGTATTGGGAAGGGAAACTTATCCTTCCATTCGGTGA
TCAGTGGTGGTTTAGGTTTTTCTTTGGATGGATCATGCCACCCAAGGTTTCTCTACTTAA
AGCCACTCAAGGTGAATACATTAGGAACTATTACCATCAAAACCATGTCATTCAGGATA
TGCTTGTTCCACTTTACAAAGTTGGAGATGCTCTTGAGTGGGTCCACCGTGAGATGGAGG
TATATCCCCTCTGGCTCTGTCCCCACCGACTCTACAGGTTGCCTCTTAAAACCATGGTTT
ATCCTGAACCAGGGTTTGAGCTGCACTGCAGGCAGGGTGACACACAATATGCTCAAATG
TACACCGATGTTGGTGTCTACTATGCTCCTGGACCTATTTTGAGGGGTGAGGTCTTTGAT
GGTGCAGAGGCAGTCCGTAAGATGGAGAGTTGGCTGATTGAAAACCATGGATTCCAGCC
ACAGTATGCTGTCTCCGAGCTGACGGAGAAGAACTTCTGGAGGATGTTTGATGGAGACC
TGTACGAGCACTGCAGAAGAAAGTACAAAGCCATTGGAACCTTCATGAGTGTGTACTAT
AAGTCTAAGAAAGGAAGGAAGACTGAGAAGGAGGTGCAGGACGCTGAGCAAGAGACA
GCTGAACTTGAGACCCCAGAAGTTGATGAGCCTGCAGATTGA

Supplemental Figure 3. The cDNA sequences of the putative *PaSSR1* retrieved from the calyx transcriptome of *P. angulata.*

ATGTCGGCTGCTAAGGCCCCTGCGGCCCCTGTTCGTTCCAAGAAGAAGATCCAGTTGGT
CGACTTTCTTCTCCAATTCAGATGGATCGTTGTTGTCTTCTTCGTCCTTCCTTTCTCGTTCC
TGTATTACTTCTCCATATATCTAGGGGATTTGAAAGCCGAGAGGAAATCTTACAAGCAG
CGTCAGGTAGAGCACTTAGAAAATGTTAAAGAGGTTGTGAAGCGTCTTGGTCAGAGGGA
TGCATCAAAGGACGGTCTTGTATGTACGGCCAGGCCTCCCTGGGTTGTCGTTGGAATGA
GAAATGTTGACTATAAACGTGCTCGTCATTTTGAAGTTGATCTTTCTAAGTTTAGAAATA
TACTTGAAATTGACAGGGAACGAATGATTGCCAAAGTTGAGCCTCTAGTCAGTATGGCG
CAAATCTCAAGGGTTACTGTCCCAATGAATCTTTCCCTCGCAGTTCTTCCTGAGTTTGAT
GATCTGACCGTTGGGGGTCTGATCAACGGCTTCGGGGTTGAAGGAAGCTCTCACATCTTT
GGATTGTTCTCCGATACTGTTGTTTCACTAGAGGTTGTTCTAGCAGATGGACGGGTTGTT
AGAGCTACAAAGGATAATGAGTACTCTGATCTTTTTTACGCTATCCCGTGGTCTCAGGGG
ACATTGGGTCTGCTTCTTTCTGCTGAGATCAAGCTTATACCAGTTAAGGAGTACATAAGA
CTTACCTACAAACCTGTAACTGGTAGTCTTAAAGAGCTTGCACAGGCTTATGCGGATTCC
TTTGCACCTAAAGACGGAGACCAGGACGATCATTCTAAAGTTCCAGAGATGGTGGAAGG
CATGATTTATAGTCCGACAGAAGGTGTTATGATGACCGGAACATATGCTTCAAAGAAAG
AAGCCAAACAAAGAGGTAACGTAATCAACACTTATGGTTGGTGGTTCAAACCATGGTTT
TACCAGCATGCTCAAACTGCGCTGAAAAGAGGGGAGTTTGTGGAGTACATTCCAACTAG
GGACTACTACCACAGGCACACAAGATCCTTGTATTGGGAAGGGAAACTGATTCTTCCAT
TTGGTGACCAGTTCTGGTTTAGGTTCCTCCTAGGATGGCTCATGCCACCCAAGATTGCTC
TGCTCAAAGCCACTCAAAGTGACTCCATCAGGAACTATTACCATGATCATCATGTCATTC
AGGATCTGCTTGTTCCTCTTTACAAAGTCGGAGATACCCTAGAGTGGGTTCACCGCGAG
ATGGAGGTATATCCCATCTGGCTCTGCCCACACAGAATTTACAAGCTGCCTGTGAAACC
AATGATCTCTCCTGAACCAGGATTCGAGGCACACAACAGGCAGGGTGACACCAAATATG
CTCAAATGTACACAGATGTGGGTGTCTACTATGTTCCTGGAGCTGTCCTGAGGGGCGAG
CCCTTTGACGGTGCAGAGAAATGCCGCCAACTTGAGCTTTGGTTGATCGAAAACCATGG
ATATCAGGCTCAGTACGCTGTCACTGAGCTATCAGAGAAAAACTTCTGGAGGATGTTTG
ATAACACCTTGTACGAGAAGTGCAGAAGAAAGTACAAAGCCATCGGAACGTTCATGAA
CGTGTACTATAAATCTAAGAAAGGAAGGAAGACGGAGAAGGAGGTGCAGGAAGCCGAG
CAAGAGAAAGCTGAACAAGATACTCCTGAAGTCGATGAGCCCGTAGAATATTAA

Supplemental Figure 4. The cDNA sequences of the putative *PaSSR2* retrieved from the calyx transcriptome of *P. angulata.*

ATGTCTGATGAAAAAGTTGCTACTGTTAGACCTAAAAGAGAAACTAGATTGGTCGATTT
TTTAGTTCAATTTAGATGGATTGTTGTTATTTTTTTTGTTTTGCCATTCTCTTGCTTGTATT
ATTTTTCTATCTATTTGGGTGATGTTAGATCTGCAAGAAAATCTGAATCTCAGAGAAAAA
AGGAACATGAGGAAAATATCAAAGAAGTTGTTAAAAGATTGGCTCAAAGAGATGCTTCT
AAAGATGGTTTGGTTTGTACTGCTAGACCACCATGGACAGTTATTGGAATGAGAAATTG
TGATTACAAAAGAGCTAGGTCATTTAAAGTTGATTTGTCTAAGTTTAGAAATATTTTGGA
AATTAACAAGGAAAGAATGGTTGCTAAAGTTGAACCATTGGTTAATATGGCTCAAATTT
CTAGAGCTACTATTCCAATGAATTTGTCTTTGGCTGTTATTGGTGAATTGGATGATTTGA
CTGTTGGTGGTTTGATTAATGGTTTTGGTATTGAAGGTTCTTCTCATATTTATGGTTTGTT
TTCTGATACTATTGTTGCTTTAGAAGTTGTTTTAGCTGATGGTAGAGTTGTTAGAGCTACT
AAAGATAATGAATATTCTGATTTGTTTTATGCTATTCCATGGTCTCAAGGTACTTTGGGA
TTGTTGGTTTCAGCTGAAATTAAATTGATTCCTGTTAAGGAATATATGAAATTGACTTAT
AAACCTGTTACTGGTAATTTGAAAGAATTGGCTCAAGCTTTTGCTGATTCTTTTGCTCCA
AGAGATGGTGATCAAGATAATCCATCTAAAGTTCCTGAATTTGTTGAAGGTATGATTTAT
TCTCCAACTGAAGGTGTTATGATGACTGGTAGATATGCTTCTACTAAAGAAGCTAAACA
AAAGGGTAATGTTATTAATTCTATTGGTTGGTGGTTTAAACCATGGTTTTATCAACATGC
TCAAACTGCTTTGAAAAGAGGTGAATTCGTTGAATATATTCCAACTAGAGATTACTGTCA
TAGGCATACTAGATCTATTTATTGGGAAGGTAAATTGATTTTGCCATTTGGTGATCAATT
TTGGTTTAGATATTTGTTTGGTTGGATGATGCCACCAAAAGTTGCAGTTTTGAAGGCTAC
TCAATCAGAATATATTAGAAATTATTATCACGACAATCATGTTATTCAAGATTTGTTGGT
TCCATTGCATAAAGTTGCTGATACTTTGGAATGGGTTCATAGAGAAATGGAAGTTTATCC
TGTTTGGTTGTGTCCACATAGAATTTATAAATTGCCTGTTAAACCAATGATTCATCCTGA
ACCTGGTTTTGAAGCTCATAAAAGACAAGGTGATACTAAATATGCTCAAATGTATACTG
ATGTTGGTGTTTATTATGCTCCTGGTGCTGTTTTGAGAGGTGAACCTTATGATGGAGCTA
AAAAATGTCATGATGTTGAGATTTTTTTGATTGAAAATCATGGTTTTCAGCCACAATACG
CTGTTACAGAATTGTCTGAAAAAAATTTTTGGAGAATGTTTGACGCAACTTTGTATCAAC
AATGTAGAAAAAAATATAAAGCTATTGGTACTTTCATGAATGTTTATTATAAATGTAAA
AAAGGTAAAAAAACTGAAAAAGAAGTTCAAGAAGCTGAACAAGAAAAAGCTGAATTGG
AAACTCCTGAAGTTGATGAACCTGCTGATTAA

Supplemental Figure 5. The optimized sequences of *Pa24ISO* following the codon usage preference of *Saccharomyce cerevisiae.*

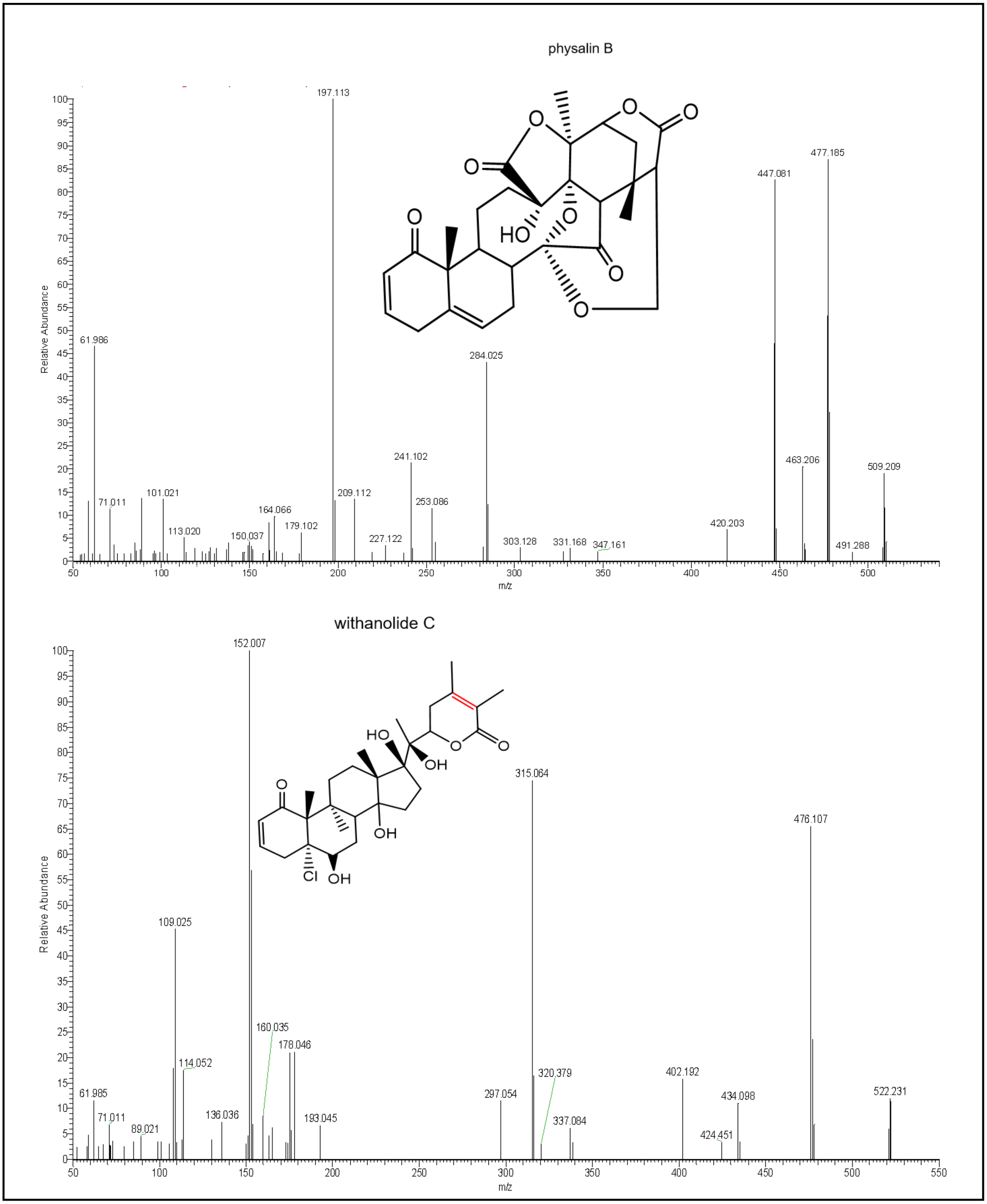

Supplemental Figure 6. MS/MS spectra of physalin B and the putative withanolide C extracted from *Physalis angulata.*

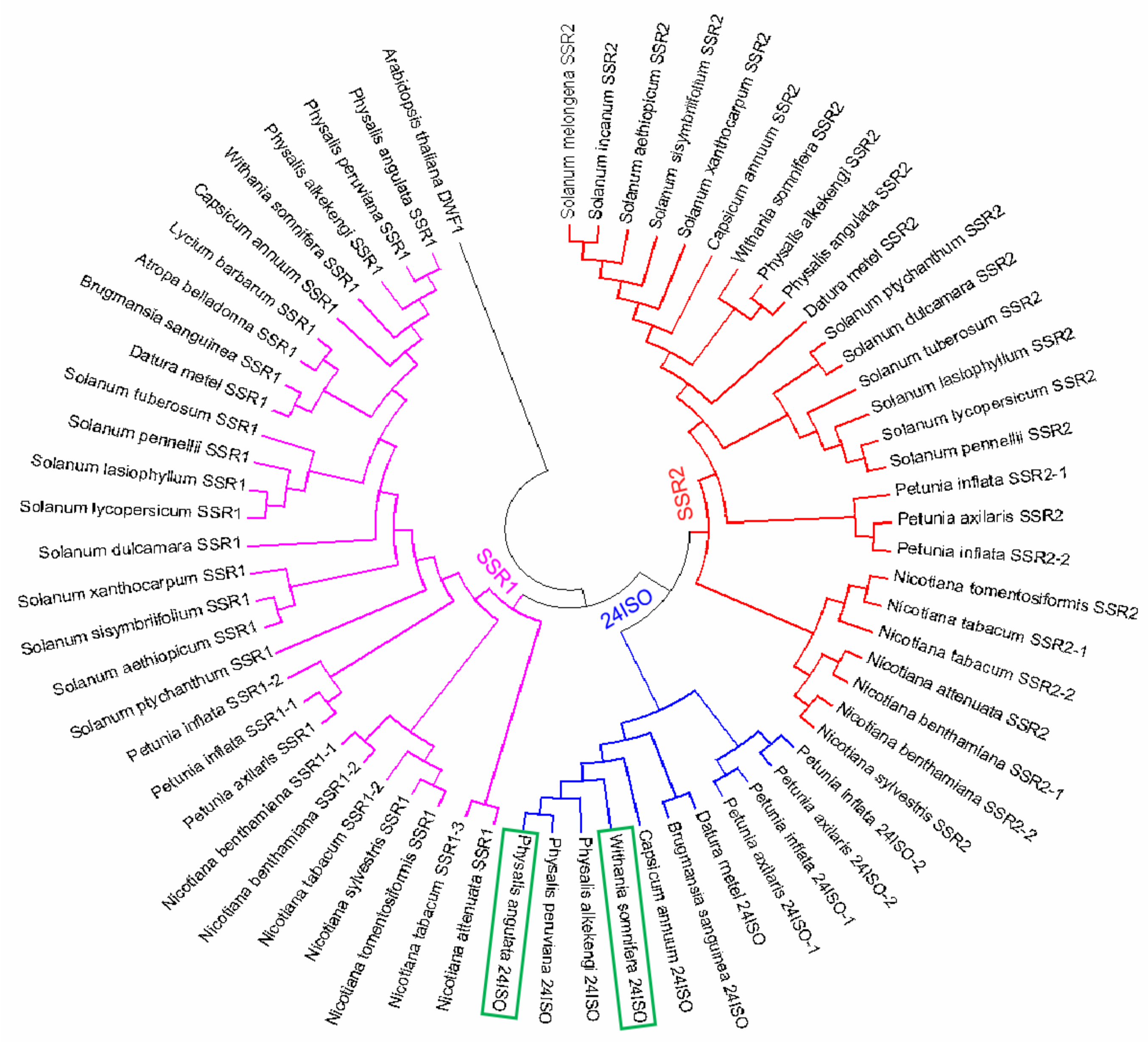

Supplemental Figure 7. Phylogenetic analysis of Pa24ISO with the previously reported orthologs of 24ISO, SSR1 and SSR2. The tree was divided into three distinct clades, which included the SSR1 (magenta), SSR2 (red), and 24ISO (blue) groups. Phylogenetic relationship was inferred using the maximum likelihood method in Mega7 (based on 1,000 replicates). Arabidopsis thaliana DWF1 was used to root the tree. Ws24ISO and Pa24ISO grouped into the 24ISO clade, and they are marked in a green rectangle.

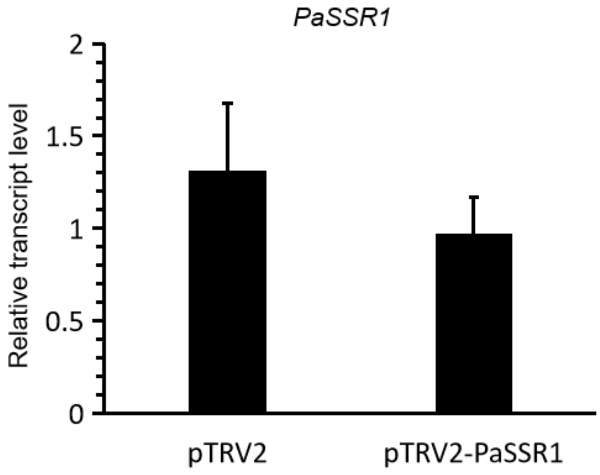

Supplemental Figure 8. Real-time PCR analysis of the *PaSSR1*expression in the *Pa24ISO*-silenced leaves (pTRV2-Pa24ISO) relative to the empty vector control (pTRV2).

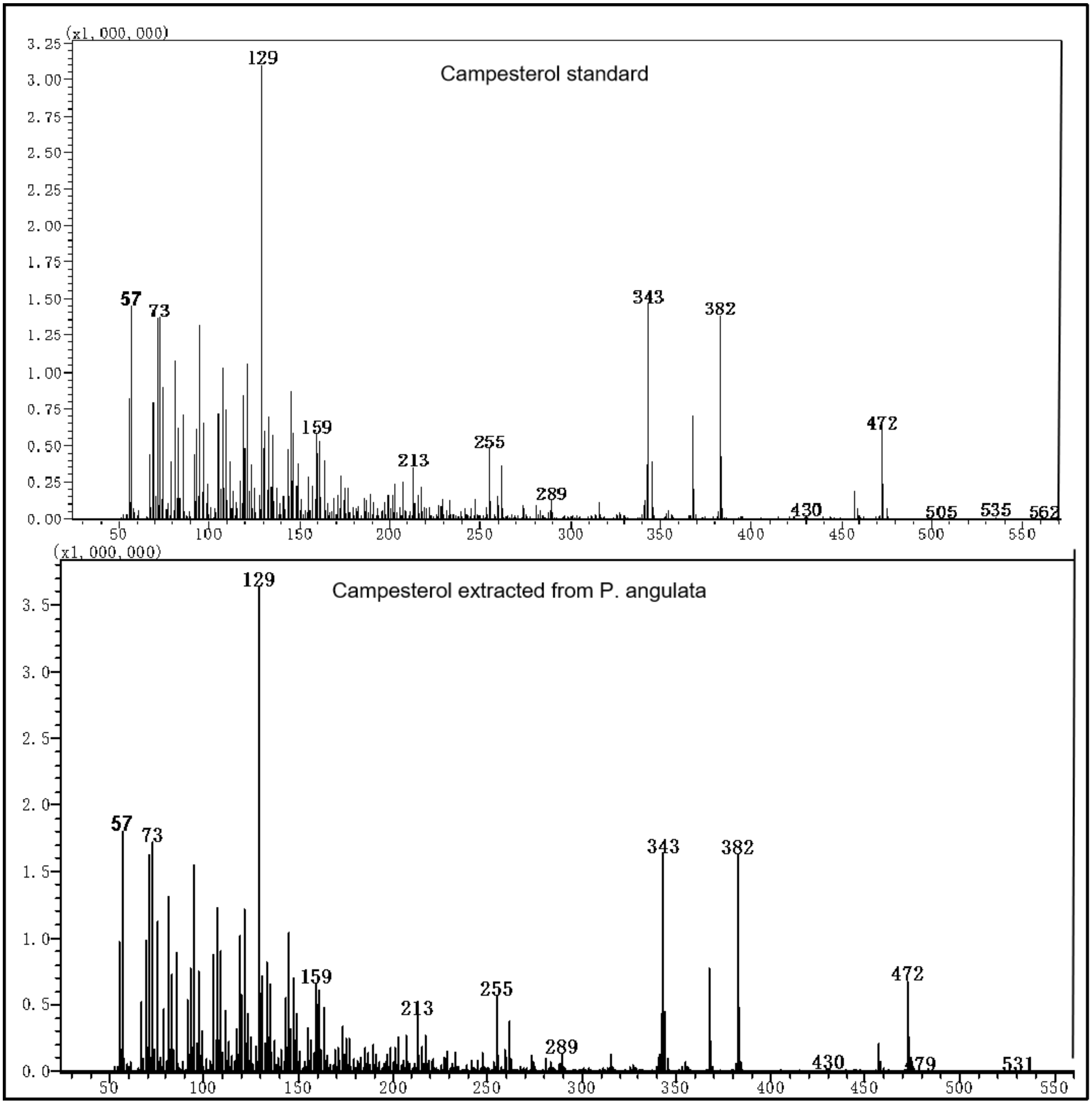

Supplemental Figure 9. MS/MS spectra of the campesterol product extracted from the *P. angulata* leaves in comparison with the campesterol standard.
